# Ethical perspectives on recommending digital technology for patients with mental illness

**DOI:** 10.1186/s40345-017-0073-9

**Published:** 2017-02-07

**Authors:** Michael Bauer, Tasha Glenn, Scott Monteith, Rita Bauer, Peter C. Whybrow, John Geddes

**Affiliations:** 1 Department of Psychiatry and Psychotherapy, Universitätsklinikum Carl Gustav Carus, Technische Universität Dresden, Fetscherstr. 74, 01307 Dresden, Germany; 2 ChronoRecord Association, Inc., Fullerton, CA 92834 USA; 3 Michigan State University College of Human Medicine, Traverse City Campus, 1400 Medical Campus Drive, Traverse City, MI 49684 USA; 4 Department of Psychiatry and Biobehavioral Sciences, Semel Institute for Neuroscience and Human Behavior University of California Los Angeles (UCLA), 300 UCLA Medical Plaza, Los Angeles, CA 90095 USA; 5 Department of Psychiatry, University of Oxford, Warneford Hospital, Oxford, OX3 7JX UK

**Keywords:** Mental illness, Digital healthcare, Ethics, Digital economy, Privacy

## Abstract

The digital revolution in medicine not only offers exciting new directions for the treatment of mental illness, but also presents challenges to patient privacy and security. Changes in medicine are part of the complex digital economy based on creating value from analysis of behavioral data acquired by the tracking of daily digital activities. Without an understanding of the digital economy, recommending the use of technology to patients with mental illness can inadvertently lead to harm. Behavioral data are sold in the secondary data market, combined with other data from many sources, and used in algorithms that automatically classify people. These classifications are used in commerce and government, may be discriminatory, and result in non-medical harm to patients with mental illness. There is also potential for medical harm related to poor quality online information, self-diagnosis and self-treatment, passive monitoring, and the use of unvalidated smartphone apps. The goal of this paper is to increase awareness and foster discussion of the new ethical issues. To maximize the potential of technology to help patients with mental illness, physicians need education about the digital economy, and patients need help understanding the appropriate use and limitations of online websites and smartphone apps.

## Background

Today there are many sources of big data in medicine beyond those created directly by physicians in electronic medical records (EMR). Data may be linked from imaging, pharmacy records, laboratory data, ‘omics data (large-scale genomic, metabolomic, and proteomic datasets), and administrative claims from government and private insurers (McKinsie 2011; Monteith et al. 2016a). In the future, IBM predicts that the majority of medical data will be created by patients and non-providers from health apps, patient monitoring, and from behavioral data based on the tracking of daily digital transactions (Slabodkin 2015). Important features of big data are massive size, heterogeneity, uneven quality, and the need for sophisticated automated techniques to find meaning. Already, clinical data from many provider systems are being shared in large regional or national databases to improve consistency of care, and to facilitate a wide range of medical research that increasingly involves commercial organizations (Powles 2016; IBM 2016). Both the adoption of digital apps and monitoring devices, and use of analytics on big data from diverse sources are considered the key aspects for improving healthcare and increasing cost-efficiencies (WEF 2016).

But the growth of big data and data sharing may also result in serious non-medical and medical issues for patients. The same big data technologies and analytical techniques used in medicine are also used for commercial purposes. The behavioral data acquired from the continual tracking of digital activities are sold in the secondary data market and used in algorithms that automatically classify people (Executive Office 2016; FTC 2016a). These classifications may affect many aspects of life including credit, employment, law enforcement, higher education, and pricing. Due to errors and biases embedded in data and algorithms, the non-medical impact of the classifications may be damaging to those with mental illness who already face stigmatization in society (Monteith and Glenn 2016). There are also potential medical risks to patients associated with poor quality online information, self-diagnosis and self-treatment, passive monitoring, and the use of unvalidated smartphone apps.

The goal of this paper is to increase understanding and promote discussion of the ethical issues of the digital economy that affect the treatment of patients with mental illness. Without an understanding of the digital economy, physician recommendations to patients to use technology may inadvertently lead to harm. Before discussing the ethical issues, a brief background on the digital economy, data privacy, and societal pressure to disclose information is provided.

## Digital economy

The impact of big data on healthcare is part of the ongoing digitization of all major industries. Personal data are viewed as the fundamental and transformative asset class of the new digital economy and the basis for analytic decision-making (WEF 2012, 2016). Massive amounts of personal data are created and tracked from all aspects of life that involve technology, including routine daily activities such as using the Internet, social media, cell phones, smartphones, email, credit and debit cards, customer loyalty cards, posting pictures online and making mobile payments. Increasingly, large amounts of machine-generated data are produced by sensors, video cameras, license plate readers, GPS systems, E-ZPass, RFID (radio frequency identification) devices, and fitness trackers (IDC 2014). Metadata (data about data) is collected to provide context. Modern tracking techniques include sophisticated browser fingerprinting, and cookie syncing (user ID sharing) between trackers (Englehardt and Narayanan 2016). In the past, it was only profitable to collect personal data about the rich and famous (Goldfarb and Tucker 2012). The costs of data capture, storage, and distribution are now so low that it is profitable to collect personal data about everyone.

Personal data are collected by data trackers, combined with other data, analyzed, and re-sold as data products by data brokers (Martin 2015; GAO 2013; WEF 2012). A standard business model for online companies that provide free services, such as search engines and medical sites, is to track activities for behavioral advertising and sell this personal data to third parties (Goldfarb and Tucker 2011; Stark and Fins 2013; Rosenberg 2016). Commercial, governmental, and academic firms who purchase data products often recombine and re-analyze the data. Digital copies of data products can be sold endlessly. Personal data are valuable because it provides information about a person’s behavior based on the details of daily activities, thoughts, and personal connections (Pentland 2012). The value of personal data increases as the number of connections with other datasets increases.

Much of the personal data is sensitive information that is voluntarily shared by individuals, their friends, and their family (Fairfield and Engel 2015). Although metadata does not contain content, it often provides information just as sensitive as content, such as documenting regular calls to a psychiatrist’s office. Data from sources that appear harmless and unrelated may be combined to detect highly sensitive information, such as predicting sexual orientation from Facebook Likes (Kosinski et al. 2013). Firms are combining data from credit card purchases, lifestyle factors, Internet searches, and social media to recruit for clinical trials without accessing medical records (Walker 2013). Many individuals are not aware of activity tracking, and the buying and selling of their personal data (FTC 2014). Online personal data contain many errors, yet digital copies exist at different locations, making it nearly impossible to correct or permanently delete the data (PCAST 2014).

The collected data based on tracking behaviors enable automated decision-making, such as consumer profiling, risk calculation, and measurement of emotion. These algorithms broadly impact our lives in education, insurance, employment, government services, criminal justice, information filtering, real-time online marketing, pricing, and credit offers (Yulinsky 2012; Executive Office 2016; Pasquale 2015). Decision-making independent of human involvement may perpetuate long-standing inequalities and exclusions, due to errors and human biases embedded in data and algorithms (Executive Office 2016; FTC 2016a; PCAST 2014). A report from US Executive Office warns that “big data could enable new forms of discrimination and predatory practices” (Executive Office 2014), which is of particular concern to those with mental illness (Table 1). The details of most commercial and governmental algorithms are hidden from public view, leaving the public little recourse to challenge decisions (Pasquale 2011; Kerr and Earle 2013).

**Table 1:** Examples of automatic classification of people based on big data in the US

Area	Goal of automation	Negative consequence
Criminal justice	Predict involvement in violent crime	Automated predictions of future bad behavior or guilt by association, in high-crime areas (Robinson et al. 2014)
Employment	Display job openings based on user profiles	Job opportunities not offered based on traditional biases (Sweeney 2013; Savage 2016)
Employment	Automate job applicant screening^a^	Individuals flagged as potentially having stigmatized or expensive disease based on algorithm (Rosenblat et al. 2014)
Employment	Employer sponsored wellness programs include fitness trackers^a^	Preferential treatment and promotions to those who participate (Rosenblat et al. 2014; Christovich 2016)
Financial	Include health and lifestyle habits in non-traditional, credit-related scoring algorithms	Decreased credit or higher interest rates on credit cards for the sick (Dixon and Gellman 2014; Robinson et al. 2014)
Higher education	Predict good candidates for higher education	Opportunities not offered based on traditional biases. (FTC 2016a)
Insurance	Determine health status without physicals	Higher life insurance rates for those at higher risk (Batty et al. 2010; Robinson et al. 2014)
Online commerce	Conditional (dynamic) pricing based on user profiles	Higher prices for those living in poor areas with less retail competition (Valentino-Devries et al. 2012; Acquisti and Varian 2005). MAC users shown more expensive goods than PC users (Mattioli 2012)
Online commerce	Offer credit online based on user profiles	No credit offers from leading institutions to those with poor credit (Fertik 2013)
Online information seeking	Provide news and information based on user profile	Reinforce prejudices and increase insularity (Pariser 2011)

^a^About 56% of US population covered by employer-based health insurance (US Census 2016)

Algorithms based on big data are also used by criminals to target potential victims, and some people with mental illness may be especially susceptible (Monteith and Glenn 2016). Factors that increase vulnerability to online fraud include intermittent Internet use, less familiarity with technology (Sheng et al. 2010; Downs et al. 2007), high impulsivity, low attention to online cues (Mayhorn et al. 2015), and cognitive impairment (Claycomb et al. 2013).

## Data privacy

One consequence of the digital economy is a loss of personal privacy (Wigan and Clarke 2013). According to Eric Schmidt, Executive Chairman of Alphabet (Google’s parent company), “We know where you are. We know where you’ve been. We can more or less know what you’re thinking about.” (Saint 2010). All thoughts, ideas, pictures, emotions, priorities, and prejudices that are publicly disclosed on social media are sold (Claypoole 2014). Many experts, including the US FBI director and the CEO of Facebook, tape the camera on their laptops and smartphones to prevent video surveillance (Hern 2016). The technologies of cloud computing and mobile devices create more challenges to privacy (Benkler 2016), and big data enables very large-scale breaches (Matwin 2013). Soon, almost everything will contain an embedded chip (Internet of Things), with Cisco estimating that 37 billion intelligent things will be connected and communicating by 2020 (Evans 2013). These connected things can be controlled remotely without human intervention, collect data, make decisions, create new privacy threats (Schneier 2016; Sarma 2015), and further erode privacy in the home (Friedland 2015).

The two primary approaches to online privacy protection are notice and choice (online individual consent at websites or apps) and anonymization, but these approaches to online privacy are not effective (PCAST 2014). Individuals rarely read online consent forms (PCAST 2014). The average person would need 201 h to read the privacy policies for the websites they visit in a year (McDonald and Cranor 2008). De-identification (anonymization) techniques are increasingly defeated with high-dimensional big data (PCAST 2014). Online privacy tools are confusing and ineffective for most people (CMU 2011). Even though privacy in the era of big data is very complex, changes to the legal framework are coming, including the general data protection regulation (GDPR) to be implemented by 2018 for the EU (EU News 2016), and the EU-US Privacy Shield (EU-US 2016).

## Societal pressure to disclose information

At the same time that technology is making it easy to collect massive amounts of personal data, commercial organizations are promoting self-revelation to make greater profits, and governments are promoting sharing to improve healthcare for the greater good.

Businesses that profit from collecting, analyzing, and selling personal data study online behavior and incorporate measures to encourage disclosure (Acquisti et al. 2015; Claypoole 2014; Google 2016a). People divulge information online because they are susceptible to manipulations that promote disclosure (Acquisti et al. 2015), and because it is intrinsically rewarding (Tamir and Mitchell 2012). Websites are designed with trust-building techniques that generate a sense of community and facilitate sharing, such as providing the perception of control (Siau and Shen 2003; Luo and Najdawi 2004; Brandimarte et al. 2013). Default privacy settings have a huge impact since these are rarely changed (Gross and Acquisti 2005; Acquisti et al. 2015). Reciprocity, or when the questioner offers information first, will increase responses to personal questions even when the questioner is a computer (Barak and Gluck-Ofri 2007; Fogg and Nass 1997; Harris 2016). Social media websites use reciprocity to expand contact lists and also foster activities that provide social approval such as tagging photos (Harris 2016). Other measures to promote disclosure include site registration, sweepstakes that require registration (Neus 2000), and pop-up forms to collect data before allowing task completion (Conti and Sobiesk 2010). Additionally, many respected and well-publicized leaders of technology companies are champions of changing societal norms about privacy (Johnson 2010; Noyes 2015; Gralla 2010).

Public health organizations in the US and UK promote digital tools as a means to engage patients as active participants and empower patients with information (Mostashari 2013; Gov.UK 2016). In the US, HHS envisions the use of mobile devices for continuous patient monitoring (Mostashari 2013), including for behavioral health (Wong 2013). Mobile apps are seen as a means to promote healthy lifestyles and behavioral changes (Webb et al. 2010; Dennison et al. 2013), and digital engagement is viewed as a positive attribute that will decrease healthcare costs for society (Lupton 2013). Major healthcare initiatives involve the creation of large national cohorts like the UK Biobank (0.5 million people) and the US Precision Medicine Initiative (goal of 1 million people) (Biobank 2016; White House 2015). These projects strongly emphasize data sharing, and many have plans to include mobile devices for patient monitoring and promotion of healthy behaviors (PMI 2015).

With the emphasis on data sharing by government and industry, privacy is often portrayed as an impediment to progress such as to achieving data-driven advances in healthcare (Cairns 2015; Goldfarb and Tucker 2012; Sarpatwari and Gagne 2016). Privacy regulation is often described as stifling technological innovation (Ruoff 2016). Yet, despite the pressure to disclose, people still want privacy. In the US, privacy remains important to people of all ages, including young adults aged 18–24 years (Hoofnagle et al. 2010). Ninety-two percent of Americans want the right to delete all online information (Turow et al. 2009). There is a special unease relating to disclosure of medical data. Many in the US and UK remain concerned about the privacy of data in the EMR, would like to limit sharing (Kim et al. 2015; Schwartz et al. 2015; eTRIKS 2016) and especially of sensitive data (Caine and Hanania 2013; Flynn et al. 2003; Snell 2017). Between 27–54% of patients may withhold information from a physician due to technology-related privacy concerns (Fair Warning US 2011; Fair Warning UK 2012; California HealthCare 2010). Most teenage patients with chronic illness do not disclose their health information on social media (van der Velden and El Emam 2013). In a recent international study of patients with bipolar disorder, the reason many looked online for information was because they incorrectly thought they would be anonymous (Conell et al. 2016).

## Ethical issues

Given the opaque nature of the digital economy and the disruptions associated with rapidly evolving technological change, new ethical issues are arising in psychiatry from the use of technology. The classification of individuals based on big data may have long-lasting and negative non-medical impacts (Executive Office 2016; FTC 2016a). The use of unvalidated apps, medical websites with poor quality information, or self-diagnosis and self-treatment may lead to medical risks, including a delay in seeking professional help (Ryan and Wilson 2008; Armontrout et al. 2016). Traditional societal concepts of what data are public versus private data, and medical versus non-medical are blurring (Tene and Polonetsky 2013; Monteith and Glenn 2016; Friedland 2015). Without addressing the new ethical issues, physicians may inadvertently harm patients with mental illness by recommending the use of technology. To discuss these ethical issues, several questions will be posed.

Issue 1: Should physicians recommend digital technology when patients lack technical skills and understanding of the digital economy?

Patients vary greatly in access to digital technology, technical skills, ability to safely use the Internet, and understanding of the digital economy. Disparities in Internet access, referred to as the “digital divide,” may be due to socioeconomic factors including income (Hilbert 2014), education (Cruz-Jesus et al. 2016), age (Friemel 2016), and the telecommunications infrastructure (ITU 2014). Although access has dramatically increased internationally over the last decade, Internet and smartphone use remains much lower for those with mental and physical disabilities and the elderly than for the general public (Choi and DiNitto 2013; Klee et al. 2016; Miller et al. 2016; Friemel 2016). Internet access for the poor may be intermittent and unreliable (Gonzales 2016). The digital divide is now evolving to reflect differences in technical skills, online literacy, and usage patterns, with less educated people spending more time on entertainment and less time information seeking (Büchi et al. 2015; van Deursen and Van Dijk 2014).

It is often mistakenly assumed that younger people are universally competent with technology. However, there are considerable differences in online skill levels among those who grew up surrounded by digital technologies (Hargittai 2010; ICILS 2014; Selwyn 2009). Modern digital technologies such as smartphones and video games are so widespread because they can be easily used by people without a technical background. Concepts of digital competency have evolved from an understanding of how technology works to being capable of using digital devices to achieve goals and solve tasks. People are not good at self-rating technical skills (Conell et al. 2016; Ivanitskaya et al. 2006), and even a technically skilled person who uses devices properly may not understand the increasingly interconnected digital economy.

There is no obvious way for the physician to know if a patient has sufficient knowledge of the digital economy to use technology wisely. The risk of digital data generated from the use of a smartphone app or Internet activities being used against a patient’s interest outside of medicine is real. Furthermore, there will always be significant inequalities in access and skills since technology keeps evolving, with industry creating new products and services (Hilbert 2016; Arthur 2010). Constant technological progress will always be accompanied by disparities in the diffusion and adaption of the new innovations.

Issue 2: Can physicians ignore patient use of digital technology?

One major benefit of the Internet is the abundance of medical information, and about 3/4 of Internet users in Europe and the US seek medical information (Andreassen et al. 2007; Pew Research 2013; Bauer et al. 2016). The quality of information about mental illness on the websites ranked highly by general search engines is generally good but does vary (Grohol et al. 2014; Reavley and Jorm 2011; Monteith et al. 2013). Searching for medical information is not easy. Consumers often judge medical websites by the visual appearance (Fogg et al. 2003; Robins et al. 2010), and may accept the first answer they receive (de Freitas et al. 2013; Conell et al. 2016). In a recent study, it was hard to get answers to general mental health questions from the well-organized NIMH website (Crangle and Kart 2015). Websites usually contain introductory information about a disease, but patients often have multiple medical and psychiatric diagnoses, long-standing illness, take numerous medications, and are looking for answers about their personal situation (Conell et al. 2016; Miller 2007). Most patients do not discuss information found online with their physicians (Conell et al. 2016; Chung 2013).

The frequency of online self-diagnosis is increasing rapidly, and may be particularly attractive to those suspecting mental illness because of the stigma, a desire for privacy, and a need to save money. One-third of adults in the US use Internet resources to self-diagnose (Kuehn 2013), and there are 50 million uses yearly of the iTriage app for symptom checking and provider selection (Aetna 2013). Many websites contain symptom checkers for mental disorders. For example, the UK NHS offers online self-assessments for sleep, mood, depression, and money worries (NHS Tools 2016), and the US VA for alcohol abuse, depression, PTSD, and substance abuse (VA 2016). Symptom checkers are also found on smartphone apps (Shen et al. 2015; Lupton and Jutel 2015) and direct-to-consumer (DTC) pharmaceutical advertising websites where legal (Ebeling 2011). Patients may also receive targeted online advertising for DTC genetic and other laboratory testing (NLM 2016; AACC 2015). Diagnosis is routinely discussed in some online mental health communities (Giles and Newbold 2011). However, a study of 23 symptoms checkers (online and apps) found that the diagnostic and triage advice across a wide range of medical diagnoses was often inaccurate (Semigran et al. 2015).

Some patients who self-diagnose may then proceed to self-treat. Virtually every prescription drug can be purchased from an online pharmacy (Orizio et al. 2011). Drugs prescribed for psychiatric disorders are a leading class of drugs sold at rogue online pharmacies that do not require a prescription (Leontiadis et al. 2013). Websites for many rogue pharmacies are professionally designed, contain false quality seals, and cannot be differentiated from legitimate pharmacies solely by appearance (Monteith et al. 2016b). About 1/3 of patients with mental illness take supplement products, which are often self-selected, purchased online, associated with false advertising claims and quality problems, and may interact with prescribed medications or other supplements (OIG 2012; Bauer et al. 2015; Wu et al. 2007; O’Connor 2015). In 2015, there were over 47,000 mental health apps on sale to US consumers offering many functions (IMS 2015). Most of these apps were not validated, and only a few were tested, primarily in small, short-term pilot studies (Donker et al. 2013; Anthes 2016).

Some health websites use fraudulent tactics or promote illegal or dangerous activities. For example, Lumosity was fined for unfounded claims of cognitive enhancement from online games and apps (FTC 2016b). Some online self-tests for Alzheimer’s disease are not valid or reliable, and do not follow ethical norms for medical interventions (Robillard et al. 2015). Drugs of abuse are readily available online such as opioids (Bert et al. 2015), stimulants (Ghodse 2007), and hallucinogens (Barratt et al. 2014). Other websites intentionally promote dangerous behavior including suicide (Luxton et al. 2012) and anorexia (Borzekowski et al. 2010). Some patients even build do-it-yourself (DIY) medical devices from instructions available online, including dangerous DIY transcranial direct current stimulation devices (Greene 2016; Wurzman et al. 2016).

Physicians should assume that all patients will use digital technology at some point in the diagnosis and course of a chronic psychiatric illness. It is notable that the many of the same instruments used by physicians to screen and monitor mental illness are now available online at no cost to patients, including the scoring cutoffs. For example, many instruments are available for depression screening including the PHQ-9, Beck Depression Inventory, Duke Anxiety-Depression Scale (DADS), and the Edinburgh Postnatal Depression Scale (USPTF 2015; VA 2016; UCSF 2013; Duke University 2016; Kerr and Kerr 2001). The public now has access to physician screening tools without the knowledge and experience to interpret the results.

While many patients are not thinking about privacy while searching online (Conell et al. 2016; Libert 2015), a study of over 80,000 health-related websites found that over 90% of the websites sent information to third parties, with 70% of these including specifics on symptoms, treatments, and diseases (Libert 2015). Patients need basic information to use digital technologies with the least risk of harm, including guidance to help clarify the limits of self-diagnosis and self-treatment. A list of a small number of recommended websites should be provided to patients (Monteith et al. 2013; Conell et al. 2016).

Issue 3: Do physicians understand mental state monitoring by commercial organizations?

With the coming of the Internet of Things, the next evolutionary step in computing is widely seen as computers reading human emotions (Pantic et al. 2007; Zeng et al. 2009; Cambria 2016). With this vision, instead of computers and devices, there will be human-centered artificial intelligence-based cognitive assistants that understand natural language, read emotions from facial expressions, voice and text, and become essential helpers throughout the day (Pantic et al. 2007; Ebling 2016; Google 2016b; Lardinois 2016). The recognition of emotion will be based on be multimodal, context-dependent systems, including facial expression and voice data (Pantic et al. 2007; Zeng et al. 2009) (Table 2). With a human–computer interface based on automated reading of emotion, users will require fewer technical skills. Personalized assistants are envisioned in medicine for both physicians and patients (Sutton 2016; Ebling 2016). There is a huge investment by the technology industry in emotion recognition. Apple, Facebook, Google, Microsoft, IBM, and Samsung were all recently awarded or applied for US patents related to inferring mood and emotion using online and smartphone data (Glenn and Monteith 2014; Brachman 2014; Kleinman 2016; Barron 2016). Today, commercial organizations and governments are routinely using algorithms based on the big data collected from the daily digital transactions to predict behavior, mental state, and to categorize and profile people (Pasquale 2015).

**Table 2:** Examples of technologies involved in automated emotion recognition

Technology	Description
Body language and gesture recognition	Recognition of meaningful body movements involving the fingers, hands, face, head or body (Mitra and Acharya 2007; Kleinsmith and Bianchi-Berthouze 2013)
Facial expression analysis	Measurement and interpretation of facial expressions (Zeng et al. 2009; Sariyanidi et al. 2015)
Facial recognition	Recognition of human faces, including if background clutter and variable image quality (Zhao et al. 2003; McPherson et al. 2016)
Natural language processing	Automatic extraction of meaning from human languages, both text and speech, requiring ambiguity resolution (Nadkarni et al. 2011)
Pattern recognition	Automated recognition, description, and classification of patterns, often involving statistical classification and neural networks (Jain et al. 2000)
Sensors	Identification of emotion using physiological signals such as heart rate, breathing, skin conduction, physical activity (Calvo and D’Mello 2010; Jerritta et al. 2011; Sun et al. 2010)
Sentiment analysis	Binary classification of subjective opinions in text such as positive versus negative, like versus dislike (Liu 2010)
Smartphone usage patterns	Identification of mood based on measures such as number and duration of incoming/outgoing calls; outgoing text messages, app usage (LiKamWa et al. 2013; Faurholt-Jepsen et al. 2016)
Speech emotion recognition	Recognition of the emotional content of human speech (El Ayadi et al. 2011; Zeng et al. 2009)
Speech recognition	Identification and understanding of human speech, converting into text or commands (Meng et al. 2012; Xiong et al. 2016)

Academic research from various areas including computer science, linguistics, and psychology are using publicly available datasets from social media to predict mental state, including depression (Resnik et al. 2015), suicide risk (De Choudhury et al. 2016), psychopathy (Wald et al. 2012), psychological disorders (Dinakar et al. 2015), and severity of mental illness (Chancellor et al. 2016). Medical research is investigating passive data collection in humans for monitoring mental illness, with pilot studies completed for bipolar disorder (Faurholt-Jepsen et al. 2016; Gruenerbl et al. 2014; Karam et al. 2014), schizophrenia (Ben-Zeev et al. 2016; Wang et al. 2016), and depression (Saeb et al. 2015). Both the academic and medical research often use the same data elements as commercial behavioral profiling, creating parameters based on smartphone calls, app usage, text messages, smartphone sensor data on location, mobility, voice analysis, and the content of social media and text messages.

At first glance, the use of personal data for commercial profiling and medical monitoring purposes may look identical. But the motivation for using algorithms to define emotions or mental state for commercial organizations is to make money, not to help patients. Most algorithms used by commercial organizations are protected by trade secrets in the US so independent validation is not possible (Schmitz 2014). As shown with Google Flu Trends (flu tracking algorithm), the published results could not be replicated with publically available information (Lazer et al. 2014). Some commercial organizations have many more parameters for each person, and many more people in their stores of big data, and may imply they use refined versions of published algorithms. However, commercial organizations are not qualified or licensed to diagnose or dispense medical opinions or advice. If an algorithm from a commercial organization suggests a person has a “propensity to search for depression,” this information should not be treated as a medical fact, and should not impact one’s chance for employment, promotion, or credit (Pasquale 2015; Rosenblat et al. 2014).

The ability for algorithms from commercial organizations to recognize human emotions and mental states will keep improving in the future with the massive investment in this area. By 2020, the global investment in emotion detection and recognition technologies is expected to reach $22.65 billion (Marketsandmarkets 2016). There must be a clear distinction between the algorithmic findings from the practice of psychiatry, and commercial findings for profit, even though similar analytic approaches are used.

Issue 4: What is the message to patients when physicians recommend passive monitoring of mental health?

Patients who live with a chronic mental illness develop a set of coping skills that are specific to their disease and personal living situation. The skills will differ with the disease severity, general medical health, access to resources, cultural factors, and individual attitudes. Today, the message from physicians is that patients can learn the skills to recognize and control symptoms and participate in society. Changing this message to emphasize passive monitoring and reliance on technology will be welcomed by some patients and offer opportunities to reach those who do not respond to standard approaches. However, some patients with mental illness may prefer to develop and depend on coping skills rather than passive monitoring.

Although enjoyed by some patients, several lines of evidence suggest that passive monitoring may not be of universal interest. The demographics of smartwatch and fitness tracker users in the US general public show that 2/3 of smartwatch owners are males between ages 18 and 34, and 41% of fitness trackers users have an income about double the national average (Gustafson 2015; Lubhy 2015). In studies of passive monitoring of patients with chronic medical illness, issues reported include privacy, not feeling in control, preferring existing coping mechanisms, losing dignity, and not wanting a constant reminder of their illness (Mol 2009; Storni 2014; Schüll 2016; Coughlin et al. 2007).

Patient attitudes towards passive monitoring are also important because cooperation and participation are required, even while having symptoms. Patients must be aware of routine technological issues and actions that affect the results including battery failures, turning off the smartphone, lending the smartphone to someone else, storage location such as in a purse, configuration settings such as location tracking, camera covers, being out of cell phone range, and dropped calls (Baig and Gholamhosseini 2013; Burns et al. 2011; Aranki et al. 2014).

There is considerable concern that passive monitoring tools may inadvertently increase the stigma associated with mental illness. The concept that some individuals require passive monitoring for mental stability may be easily misinterpreted by the general public, who often associate mental illness with violence (Pescosolido 2013). The situation will become worse if passive monitoring is used as a punishment, such as for non-adherence, or to facilitate the job of healthcare workers. Consider that continuous GPS monitoring is only required in the US after the release from prison of offenders who committed the most heinous crimes (CDCR 2016; Shekhter 2010). If medicine promotes passive monitoring of the mentally ill, it is important to address the reality of stigma in society, and take measures to prevent further social discrimination.

Issue 5: Do physicians and healthcare administrators need education about the digital economy?

Physicians and healthcare administrators are a diverse group with different levels of interest in technology, but all need to have a basic understanding of the digital economy to avoid causing inadvertent harm to patients. Many are enthusiastic and regular users of technology, and are proficient at using smartphones, tablets, and apps. Some physicians see predictive algorithms based on big data from digital devices leading to dramatic improvement in patient care (Topol et al. 2015; Darcy et al. 2016). Other, especially older, physicians are not always comfortable with technology. For example, many physicians find that EMR systems are hard to use, time-consuming, and decrease the time available for patients (McDonald et al. 2014; Accenture 2015; Dünnebeil et al. 2012). While this may reflect the poor usability of some EMR products, nearly 1 in 5 US physicians employs a medical scribe who joins the doctor and patient in the examination room to enter data into an EMR system (Gillespie 2015; Gellert et al. 2015).

From a financial perspective, some view the use of widely available smartphone apps instead of traditional care and services for mental illness as a means to reduce costs. However, there is little evidence of efficacy for the numerous apps available for mental health (Shen et al. 2015; Payne et al. 2015; Huguet et al. 2016; Donker et al. 2013; Karasouli and Adams 2014; Nicholas et al. 2015; Anthes 2016).

Even enthusiastic adapters of technology may not be educated about the digital economy. It is important that physicians who recommend the use of technology to patients, and administrators who form policy for the use of technology, be aware of potential negative consequences related to tracking of personal data. Digital tools are an important and evolving part of medicine, and physicians and administrators need education with regular updates from independent sources, not vendors selling products.

Issue 6: Should individual physicians validate smartphone apps used to make treatment decisions?

Smartphone apps that provide data used for treatment decisions should be validated. The recent experience with the UK Health Apps Library underscores the challenge. Although a new app approval process is planned for 2017 (Gov.UK 2016), studies found inadequate security in 89% of 79 accredited apps tested (Huckvale et al. 2015), and unproved clinical value in over 85% of accredited mental health apps (Leigh and Flatt 2015). A certification process must confirm that an app is not only effective and has clinical value, but must also consider real-world operation, the pathway for all data collection, sharing, storage and retention, ownership, analysis and reanalysis, and validate the specific algorithm and conclusions drawn. There are numerous technical issues relating to data security, privacy, access control, encryption, error handling, data provenance, data storage, and data transmission (Kotz et al. 2016). Other key issues include the technical support structure available to maintain and upgrade the app over time, the frequency of security recertifications, and the requirements for recertification and data ownership policy if a company is sold.

An app that collects data based on hardware components or sensors needs to be certified separately for each make and model. In today’s marketplace, one typically purchases a smartphone and then purchases an app at a later date. Consider the complexity if an app collects data from sensors. The hardware manufacturer has a set of technical specifications for each sensor, which were designed to meet the needs of a consumer smartphone, not for medical monitoring. Hardware devices contain components from many suppliers, and these will vary throughout the manufacturing life of a product model. This means that two smartphones of the same make and model purchased on the same day may contain different sensors (Asif 2015, 2016; Florin 2016) and provide slightly different data that may or may not be suitable for use in medical monitoring.

While there is no obvious solution, a certifying organization that is independent of all commercial vendors is needed to validate apps that collect data used for treatment decisions. This certifying organization must have clinical and technical expertise so that physicians can reliably recommend certified products to their patients. The certifying process must be ongoing since there are rapid changes in consumer electronics with new smartphone models appearing yearly, bringing more privacy and technical challenges. The scope of the validation problem is particularly challenging for mental health apps due to their disproportionately large number. Of the disease-specific apps available to US consumers in 2015, 29% were for mental health followed by 15% for diabetes and 8% for blood and circulatory (IMS 2015). Furthermore, the number of medical app developers is growing rapidly with an estimated 58,000 worldwide (Research 2 Guidance 2016). It is also important that patients are aware that apps that are not involved in treatment decisions, and are not certified, may have errors and may not protect patient privacy.

## Limitations

This discussion only provides a limited list of the ethical challenges and does not offer specific solutions to these complex problems. Many significant issues were omitted such as how patient monitoring systems handle data that are inadvertently captured about other people such as facial images, voice recordings, and metadata (Rana et al. 2016), and new legal issues such as timeliness of response to monitoring data (Armontrout et al. 2016). Other issues that were omitted include whether health-related chatbots (automated conversational software) should deceive patients into thinking they are interacting with a human (Whitby 2014), the coming of medications with sensors for adherence monitoring (Kane et al. 2013), the monitoring of people with dementia (Niemeijer et al. 2011), and the evaluation of long-term clinical value.

The challenges related to the adoption of new technologies including operational and technical issues, and the threats of malicious hacking into every electronic device and system used by patients and providers were not included. Provider responsibility for securing medical data was not discussed even though breaches in the US involved over 113 million records in the year 2015 (GAO 2016). The productivity paradox associated with new technologies, such that increased productivity and cost savings require an expensive multiyear process reengineering effort, was omitted (Jones et al. 2012; Brynjolfsson and Hitt 1998; Katz et al. 2012). Finally, there was no discussion of automation bias (unthinking reliance on technology) in relation to patient monitoring, which may be of concern given the quality of many sensors used in smartphones and wearables (Puentes et al. 2013; Baig and Gholamhosseini 2013; Banaee et al. 2013; Burns et al. 2011; Meltzer et al. 2015; Goode 2016).

## Conclusions and future directions

In the future, physicians will have to address technology issues to provide quality care to their patients. The digital revolution in medicine offers exciting new directions for the treatment of mental illness including online psychotherapy, tools to support medication adherence, telemedicine, and research based on linked medical records. Along with these opportunities come extraordinary complex challenges to privacy and security as part of the digital economy. There are a variety of new ethical issues facing physicians in relation to recommending the use of technology. Commercial activities such as profiling of behavior and mental state pose major non-medical concerns for patients with mental illness. The use of unvalidated apps, poor quality online information, self-diagnosis and self-treatment, and unique problems with passive monitoring pose major medical concerns. To maximize the potential of technology to help patients with mental illness, physicians need education about the basics of the digital economy, and must help patients to understand the limits and benefits.
